# Following instructions from working memory: Why does action at encoding and recall help?

**DOI:** 10.3758/s13421-016-0636-5

**Published:** 2016-07-21

**Authors:** Agnieszka J. Jaroslawska, Susan E. Gathercole, Richard J. Allen, Joni Holmes

**Affiliations:** 1 School of Psychology, Queen’s University Belfast, Belfast, UK; 2 MRC Cognition and Brain Sciences Unit, Cambridge, UK; 3 School of Psychology, University of Leeds, Leeds, UK

**Keywords:** Working memory, Following instructions, Enactment, Self-performed task, Action advantage

## Abstract

Two experiments investigated the consequences of action at encoding and recall on the ability to follow sequences of instructions. Children ages 7–9 years recalled sequences of spoken action commands under presentation and recall conditions that either did or did not involve their physical performance. In both experiments, recall was enhanced by carrying out the instructions as they were being initially presented and also by performing them at recall. In contrast, the accuracy of instruction-following did not improve above spoken presentation alone, either when the instructions were silently read or heard by the child (Experiment 1), or when the child repeated the spoken instructions as they were presented (Experiment 2). These findings suggest that the enactment advantage at presentation does not simply reflect a general benefit of a dual exposure to instructions, and that it is not a result of their self-production at presentation. The benefits of action-based recall were reduced following enactment during presentation, suggesting that the positive effects of action at encoding and recall may have a common origin. It is proposed that the benefits of physical movement arise from the existence of a short-term motor store that maintains the temporal, spatial, and motoric features of either planned or already executed actions.

The ability to retain and implement instructions is a common feature of many everyday activities, including following directions to unfamiliar locations, taking the correct dose of medication at the appropriate time, and remembering items from a shopping list. There is accumulating evidence that performing instructions substantially improves the accuracy of remembering them (Allen & Waterman, 2015; Gathercole, Durling, Evans, Jeffcock, & Stone, 2008; Yang, Gathercole, & Allen, 2014; Yang, Allen, & Gathercole, 2015), a finding that has important implications both for current theory and for practical situations in which accurate instruction-following is vital. Two experiments are reported that investigate the possible causes of these action-based benefits in children’s abilities to follow simple instruction sequences in working memory.

Working memory is the cognitive system of temporary storage supporting many complex cognitive activities in everyday life (e.g., Ma, Husain, & Bays, 2014; Miyake & Shah, 1999). There are several alternative models of working memory, and a common feature of many is limited-capacity storage combined with an attentional mechanism that enhances this capacity (e.g., Cowan, 2005; Luck & Vogel, 2013; Oberauer, 2002; Oberauer, Süß, Wilhelm, & Wittman, 2003; Shipstead, Lindsey, Marshall, & Engle, 2014). Investigations of the involvement of working memory in following instructions have been largely guided by the multicomponent model developed by Baddeley and Hitch (1974). This model consists of a central executive responsible for attentional control, two specialized stores for the retention of verbal and visuospatial information (the phonological loop and visuospatial sketchpad, respectively), and a multimodal episodic buffer that integrates information both within working memory and across longer term memory systems (Baddeley, 2000, 2012).

Abilities to follow verbal instructions have been linked with the first three of these subcomponents. In experiments with adult participants, Yang et al. (2014) found performance of sequences of manual actions following written instructions to be disrupted by concurrent tasks known to impair the central executive, phonological loop, and visuospatial sketchpad. Individual differences studies of children have also established that verbal complex memory span measures associated with the attentional executive control of working memory are closely linked with abilities to follow verbal instructions, such as “Touch the white bag and then pick up the yellow ruler” (Gathercole et al., 2008; Jaroslawska, Gathercole, Logie, & Holmes, 2016), and “Point to the picture at the top of page three and copy it twice” (Engle, Carullo, & Collins, 1991). Together, these results suggest that the retention and execution of verbal instructions is supported by working memory. One possibility is that the verbal content of the instructions is maintained in the phonological loop, supplemented by the storage of information from the environment in the visuospatial sketchpad, with the central executive coordinating the execution of actions through the retrieval of information from these stores.

Two key features of instruction-following are not readily accommodated by the multiple component model of working memory. First, recall accuracy has consistently been found to be enhanced when the instructions are recalled through physical performance compared to verbal repetition (Allen & Waterman, 2015; Gathercole et al., 2008; Koriat, Ben-Zur, & Nussbaum, 1990; Yang et al., 2015; Yang et al., 2014). This action advantage at recall has no obvious source within the current working memory model, as it is undiminished under dual task conditions known to disrupt its three main subcomponents (Yang et al., 2015; Yang et al., 2014). Second, physical performance at the time of presentation of to-be-recalled actions also improves subsequent recall (Allen & Waterman, 2015; Charlesworth et al., 2014; Wojcik, Allen, Brown, & Souchay, 2011), a phenomenon termed here the *enactment effect*. Similar memory benefits to subject-performed tasks (SPTs) are well-established in long-term memory paradigms with much longer delays between presentation and recall (e.g., Cohen, 1981; Engelkamp & Zimmer, 1989; Zimmer et al., 2001) that are unlikely to reflect limited capacity working memory subsystems.

The primary aim of these experiments was to identify the sources of the beneficial consequences of performing sequences of instructions both at the time of encoding and at recall in children. Two hypotheses were tested. According to the one-component hypothesis, the benefits of both action during initial presentation of the instructions and at recall originate from a motor store that temporarily maintains the temporal, spatial, and motoric features of the actions, whether planned (Koriat et al., 1990) or already executed (Smyth & Pendleton, 1989, 1990). These representations provide an additional source of information in working memory to guide recall above and beyond the phonological loop and visuospatial sketchpad. Planning to execute the action sequence at recall will therefore not enhance recall if the instructions have already been enacted at presentation because the executed action sequence will already be represented in the motor store. Preliminary evidence, consistent with these predictions, was reported in adults by Allen and Waterman (2015). In a task involving action sequences performed on abstract shapes, such as *drag the hexagon*, *flip the circle, push the moon*, the action advantage at recall was found to be greatly reduced when combined with enactment at encoding.

In contrast, according to the two-component hypothesis, the benefits of performing action sequences at presentation and at recall have distinct sources. By this account, the advantage of action at recall reflects the generation of representations in the motor store through planning to perform as proposed above, but the enactment effect at encoding originates in episodic memory. This position is consistent with the substantial body of evidence from the SPT field that the benefits of physical enactment at encoding persist beyond the time constraints of working memory. More broadly, this view also fits with other evidence that both working memory and episodic memory contribute to immediate recall (Unsworth & Engle, 2006, 2007). Potential mechanisms within episodic memory for SPT effects include rich encoding of multimodal experiences (e.g., Bäckman, Nilsson, & Chalom, 1986), the crucial role of independent encoding of motor information in inducing the effect (e.g., Engelkamp & Zimmer, 1989, 1994), and episodic integration between the action verb and the object noun (e.g., Kormi-Nouri, 1995; Mangels & Heinberg, 2006). The two-component hypothesis predicts that the benefits to recall of action at either encoding or recall should be additive because of their independent sources, and so should not interact. The primary aim of the two experiments reported here was to test these two competing hypotheses.

A second aim was to test two specific alternative explanations of the enactment advantage at presentation. Experiment 1 explored the possibility that the enactment advantage is a consequence of double exposure to the to-be-remembered information. One simple difference between purely verbal presentation of instructions and enactment-based encoding is that in the former case the instructions are presented just once (in auditory form), whereas in the other condition they are both heard and performed. The critical element in the enactment effect may therefore not be physical performance, but the fact that the instructions are experienced twice. Experiment 2 investigated whether the enactment effect is driven specifically by spatio-motoric actions at encoding rather than any type of motoric movement. This was tested by comparing performance with enactment at presentation to a presentation condition in which participants repeated aloud each step of the instruction as they heard it. Thus, a motoric response was generated, but it was articulatory rather than spatio-motoric in form.

In Experiment 1, a span-based following instruction task already shown to yield a substantial action effect at recall in both children and adults was employed (Gathercole et al., 2008; Yang et al., 2014). Children ages 7 to 9 years heard sequences of instructions to perform on objects. At presentation, the instructions were either spoken only once; performed one action step at a time by the participants as they were being spoken by the experimenter (enactment); or presented twice, with no physical performance. In the latter condition, the printed form of each step of the instruction was shown to the child for silent reading after it had been presented auditorily (orthographic presentation). A recall phase immediately followed in which participants either attempted to repeat the instruction sequence verbally or to perform it on an array of objects.

We predicted that participants would show a substantial benefit of encoding-based enactment (e.g., Allen & Waterman, 2015; Engelkamp, 1998; Zimmer et al., 2001), and of performing the action sequences at recall (Allen & Waterman, 2015; Gathercole et al., 2008; Yang et al., 2014). The finding that the effects of enactment at encoding and action at recall are independent of one another would be consistent with the hypothesis that the phenomena have separate sources in episodic and working memory. On the other hand, the finding that the benefits of action-based recall are reduced or even eliminated when the instructions are performed at the time of presentation would favor the hypothesis that a common motor store in working memory underpins both effects. With regard to the enactment effect, if it is simply due to the exposure of the instruction sequence in two alternative forms, performance in the orthographic condition should both be similar to performance in the enactment condition and lead to higher rates of recall than no enactment. However, if it is driven by the special status of action-based encoding in episodic memory, accuracy should be higher in the enactment conditions than either the orthographic or no-enactment conditions.

## Experiment 1

### Method

#### Participants

Sixty children (37 boys) attending a primary school in the South East of England participated in the study. The mean age of the sample was 8 years 10 months (*SD* = 6.57 months, min = 7 years 8 months, max = 9 years 8 months).

#### Materials

Participants were required to recall instruction sequences that increased in length in a span-type procedure. Each span consisted of a block of six trials (i.e., six to-be-recalled instruction sequences). Testing started at one action per sequence (e.g., “Touch the red ruler”), increased by one action per block (e.g., “Touch the red ruler and then pick up the yellow folder”), and was terminated after three incorrect trials in one block. The maximum span length was six actions in a sequence. The instruction sequences consisted of descriptions of actions to be performed on a set of concrete, three-dimensional props. The objects were a set of five stationary items (a ruler, an eraser, a pencil, a folder, and a box), in each of three colors (red, yellow, or blue). There were two actions: touch (e.g., “Touch the red pencil”) and pick up (e.g., “Pick up the yellow ruler”). Actions involving touching and picking up were concatenated using the adverb “then” to produce increasingly longer sequences that varied in length but not in lexical complexity. The items used in each instruction were selected at random, with the constraint that there was no repetition of color and object combination in the instruction as a whole. Performance was scored in terms of the features of all individual action phrases. All elements—actions, objects, and colors—recalled in their original position in the instruction sequence were scored as accurate. A sample instruction (“Touch the red ruler and then pick up the yellow folder”) has six features—two actions, two colors, and two objects. As an example, a sequence (“Touch the red ruler and then pick up the yellow folder”) recalled as “Pick up the red ruler and then touch the yellow folder” would yield a score of 4 because four out of six features are recalled in their correct serial order (i.e., red, ruler, yellow, folder). Perfect performance over 36 trials (i.e., six blocks of six trials) would result in a score of 378 features correctly recalled (18 features in block one, 36 features in block two, 54 features in block three, 72 features in block four, 90 features in block five, and 108 features in block six).

#### Design and Procedure

The experiment implemented a 3 × 2 repeated-measures design in which the presentation format (enactment, no enactment, orthographic) and recall type (verbal, action based) were manipulated.

Children were assessed individually in a quiet area of the school. The six versions of the instructions task were administered in a randomized order, with different conditions separated by rest intervals. Each child was seated at a table opposite the test administrator. All 15 items used in the following instructions task were positioned randomly on a 100-cm (diameter) × 68-cm (height) circular table within arm’s reach of the child. The object array was in view at all times, but the location of the props varied randomly between conditions. The laptop displaying written commands for the orthographic condition was placed directly in front of the participant, behind the array of props that was used for action recall. The presentation of both spoken and written instructions was controlled and paced by the experimenter. Performance was scored manually by the experimenter at the time of testing.

Before testing commenced, the child was asked to name and identify all objects and their labels. The instruction sequences were then read aloud by the experimenter at a rate of approximately 3 seconds per action phrase. Six experimental conditions were completed by each participant. Lists of instruction sequences were counterbalanced across conditions. In the no-enactment at encoding conditions, participants listened to instruction sequences and were restricted from manipulating any of the objects. At the end of presentation, participants were asked to recall the sequence by either performing the actions (serial action recall) or repeating them back (serial verbal recall). In the enactment at presentation conditions, instruction sequences were broken down into single action phrases (e.g., “Pick up the yellow pencil”—break—“and then touch the red box”). Children performed each action phrase immediately after its verbal presentation. For example, the experimenter read aloud “Touch the yellow ruler”, the participant enacted the command, the experimenter then read aloud the next instruction “then pick up the blue pen,” which was then enacted by the participant. Recall of instruction sequences was either action based or verbal. The performance of the actions was self-paced in the enactment conditions. In the orthographic conditions, each action phrase was spoken by the experimenter and then presented in written form on a computer screen for the participant to read. For example, the experimenter read aloud “Pick up the red box,” the participant then silently read the command from the computer screen, the experimenter then read aloud the next instruction, “then touch the blue folder,” which was then read out by the participant. For the orthographic presentation, single action instructions were presented on a 13-in. laptop screen in black font on a white background, aligned to the screen center. Each command appeared on the screen only after the action phrase was read out loud by the experimenter and was presented for approximately 3 seconds, followed by a blank screen to allow for the presentation of the following action phrase or for recall. Recall was self-paced in all conditions.

Written parental consent was obtained before testing. The study was approved and conducted in accordance with the guidelines of the Cambridge University Psychology Research Ethics Committee and the MRC Cognition and Brain Sciences Unit.

### Results

**Fig. 1:**
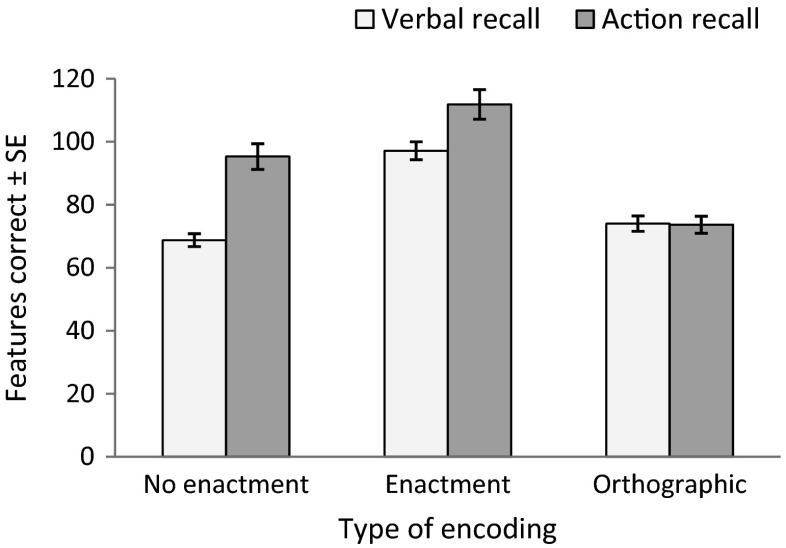
Mean total feature scores in each presentation and recall condition (error bars denote standard error)

Figure 1 shows the descriptive statistics for each of the six conditions of the instructions task. To test whether the benefits of performing instructions at encoding and recall are driven by a common mechanism, a 2 × 2 ANOVA was performed on the two presentation conditions (enactment vs. no enactment) as a function of recall (verbal vs. action). There was a significant main effect of presentation type, *F*(1, 59) = 62.299, *MSE* = 485.765, *p* < .001, η_p_^2^ = .514, with better performance in the enactment than the no-enactment condition. The main effect of recall, *F*(1, 59) = 32.709, *MSE* = 780.327, *p* < .001, η_p_^2^ = .357, reflected the benefits of action during retrieval. These main effects were qualified by a significant presentation type by recall interaction, *F*(1, 59) = 4.429, *MSE* = 475.532, *p* = .040, η_p_^2^ = .070. Planned pairwise comparisons revealed that enactment at encoding improved performance beyond spoken presentation when the instructions were either repeated verbally *t*(59) = 8.649, *p* < .001, *d* = 1.51, or acted during retrieval, *t*(59) = 3.585, *p* = .001, *d* = .49.

Next, the data were analyzed in a 3 × 2 ANOVA with repeated factors of presentation (no enactment, orthographic, enactment) and recall (verbal, action), to establish whether the enactment effect is a consequence of double exposure to the instruction sequences. There was a significant main effect of presentation condition, *F*(2, 118) = 63.903, *MSE* = 472.875, *p* < .001, η_p_^2^ = .520, and a main effect of recall type, *F*(1, 59) = 25.819, *MSE* = 648.420, *p* < .001, η_p_^2^ = .304. The interaction term was also significant, *F*(2, 118) = 13.449, *MSE* = 404.921, *p* < .001, η_p_^2^ = .186.

Simple main effects analyses were run to investigate the impact of encoding on different types of recall. When instructions had to be verbally repeated at test, recall in the enactment condition was significantly higher than in the other two presentation conditions (both *p*s < .001; all *p* values interpreted using the Bonferroni correction for multiple comparisons), but there was no significant difference between spoken and orthographic presentation conditions (*p* = .204). As already established, when recall was action-based performance was significantly higher when instructions were enacted rather than simply heard at encoding (*p* = .001). In addition, both enactment and spoken presentation led to significantly better action recall than orthographic presentation (*p* < .001).

Bonferroni-corrected planned comparisons established that accuracy was higher for action-based than for verbal recall when instructions were spoken, *t*(59) = 6.814, *p* < .001, *d* = 1.13, and when they were enacted, *t*(59) = 2.846, *p* = .006, *d* = .50. There was no evidence for an action-advantage in the orthographic condition, *t*(59) = .128, *p* = .898, *d* = .02.

### Discussion

Children’s memory for instructions was boosted both by enacting instructions during presentation and by performing them at recall. Although the advantage of action-based over verbal recall was present in the no-enactment and enactment conditions, its magnitude was reduced when participants carried out the instructions during their initial presentation. This interaction between physically performing instructions during presentation and at recall suggests that the two phenomena may be driven by a common mechanism. This is consistent with findings reported in adult populations by Allen and Waterman (2015). They found that the benefits of enactment during encoding were dependent on the type of recall required, possibly reflecting a beneficial role for spatio-motoric coding in working memory that can be engaged either through action planning or physical performance.

The additional exposure to instruction sequences provided by combining printed with spoken presentation of each step did not enhance recall accuracy. The beneficial effect of enacting the steps of the instruction sequence at presentation cannot therefore simply be explained in terms of the additional exposure to memory items resulting from two presentations. This is consistent with claims that the motoric aspect of enactment provides participants with an additional encoding modality which increases the specificity of the to-be-performed command and renders it easier to retrieve (e.g. Engelkamp & Zimmer, 1989, 1994).

As expected, an action advantage was obtained when instructions were spoken or enacted at encoding. Surprisingly, however, the advantage of action-based recall over spoken repetition was eliminated when spoken instructions were paired with print at presentation in the orthographic condition. One possibility is that reading each step immediately after hearing it may have prevented participants from generating action-based plans for executing the instructions in the motor store in working memory.

Experiment 2 sought to explore a further nonmotoric account of the enactment effect at presentation by testing whether a physical response at encoding rather than spatio-motoric involvement per se is crucial to the enactment effect. To do this, a new presentation condition was included in which the children repeated aloud (shadowed) each step of the instruction as they heard it. Thus, a motoric response was generated, but it was articulatory rather than spatio-motoric in form. The verbal and enactment presentation conditions employed in Experiment 1 were also retained in an attempt to replicate the interaction between the effects of physical performance at presentation and at recall. Our hypothesis was as follows. If the enactment effect is simply driven by the active production of an overt response during encoding (whether motoric or vocal), performance in both the enactment and shadowing conditions should lead to higher rates of recall than no enactment. Alternatively, if the enactment effect is driven by the specific availability of spatio-motoric encoding in working memory—for example, because of its distinctiveness—accuracy should be higher when instructions are enacted than when they are shadowed. Based on the results of Experiment 1, we predicted that there would be benefits of action at both presentation and recall, and that there would be a significant interaction between the two.

## Experiment 2

### Method

#### Participants

Fifty-two children (33 boys) attending two primary schools in the South East of England participated in the study. The mean age of the sample was 8 years 8 months (*SD* = 7.53 months, min = 7 years 5 months, max = 9 years 11 months). None of the participants had taken part in Experiment 1.

#### Materials

The following-instructions paradigm was identical to that used in Experiment 1, with one exception. The orthographic presentation was substituted with a different encoding condition. At encoding, instructions sequences were either spoken aloud by the experimenter (no enactment), spoken by the experimenter and verbally repeated by the participant (shadowing), or spoken by the experimenter and performed by the participant (enactment). In the shadowing condition, each action phrase was first spoken by the experimenter and then vocalized by the participant. For example, the experimenter read aloud “Pick up the red box,” the participant then repeated the command, and the experimenter then read aloud the next instruction, “then touch the blue folder,” which was then repeated by the participant. As in Experiment 1, recall was self-paced by participants in all conditions.

#### Design and Procedure

This study employed a 3 × 2 within-subjects design, in which the presentation format (no enactment, shadowing, enactment) and the type of recall (action, verbal) were manipulated. The dependent variable in each condition was the total number of features (i.e., actions, colors, objects) recalled correctly.

Children were visited at school and assessed individually on six versions of the following instructions task. Testing was completed during a single testing session. The six versions of the following instructions task were counterbalanced, with the different conditions separated by rest intervals. The testing procedures for enactment and no-enactment conditions were identical to those used in Experiment 1. The presentation of instructions was paced by the experimenter. In addition, the presentation of instructions in the enactment and shadowing conditions was audio recorded to allow comparison of encoding durations in these two conditions. At recall, participants were asked either to repeat the instructions (verbal serial recall) or to perform them in sequence (action serial recall).

Written parental consent was obtained before testing. The study was approved and conducted in accordance with the guidelines of the Cambridge University Psychology Research Ethics Committee and the MRC Cognition and Brain Sciences Unit.

### Results

**Fig. 2:**
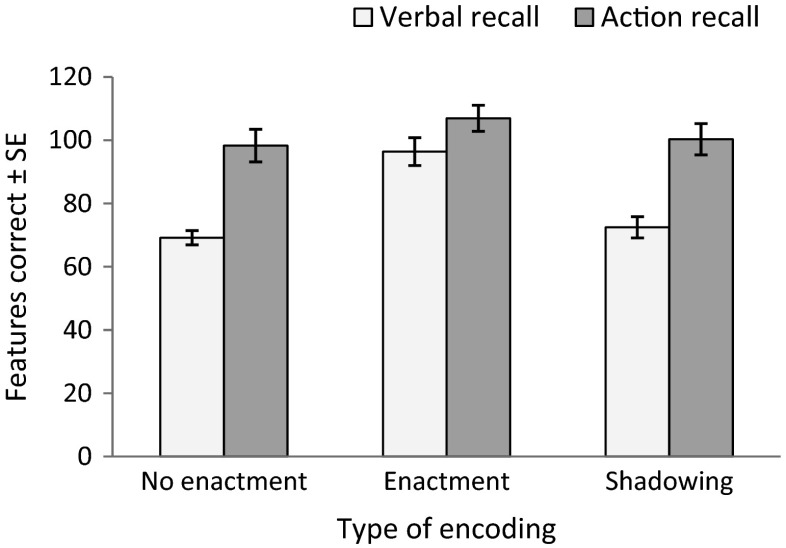
Mean number of features (i.e., action, colors, and objects) correctly recalled in each encoding and recall condition (error bars represent standard error)

Figure 2 displays average performance in each condition. The mean time to enact instructions during encoding was 2.02 seconds (*SD* = 0.04), and 1.98 seconds (*SD* = 0.05) to repeat them. This difference was not significant, *t*(51) = 1.060, *p* = .294, *d* = .172.

To test whether the benefits of action at encoding and recall are independent from one another, as in Experiment 1, a 2 × 2 ANOVA with presentation (no enactment, enactment) and recall (verbal, action) was carried out. There were significant main effects of both presentation type and recall, *F*(1, 51) = 39.091, *MSE* = 427.317, *p* < .001, η_p_^2^ = .434, and *F*(1, 51) = 37.676, *MSE* = 543.613, *p* < .001, η_p_^2^ = .425, respectively. The interaction term was also significant, *F*(1, 51) = 10.744, *MSE* = 417.572, *p* = .002, η_p_^2^ = .174. Planned pairwise comparisons revealed that enactment at encoding improved performance beyond spoken presentation when the recall was verbal, *t*(51) = 7.126, *p* < .001, *d* = 1.13, but this difference did not withstand the Bonferroni correction for multiple comparisons when recall was enacted, *t*(51) = 2.040, *p >* .0125, *d* = .26.

Next, we tested whether the enactment advantage at presentation is a result of self-production of to-be-remembered information during encoding. A repeated-measures ANOVA with factors of presentation (no enactment, repetition, enactment) and recall (verbal, action) showed a significant main effect of presentation type, *F*(2, 102) = 24.346, *MSE* = 399.369, *p* < .001, η_p_^2^ = .323. The main effect of recall was also significant, *F*(1, 51) = 47.016, *MSE* = 841.316, *p* < .001, η_p_^2^ = .480, with superior performance in the action than verbal condition. These main effects were qualified by a significant presentation by recall interaction, *F*(2, 102) = 7.634, *MSE* = 366.837, *p* = .001, η_p_^2^ = .130.

Follow-up analysis revealed that when recall was verbal, performance in the enactment condition was significantly better than performance in the other two conditions (both *p*s < .001, interpreted using a Bonferroni correction for multiple comparisons), but there was no difference between the no-enactment and shadowing presentation conditions (*p* = .869). In contrast, when instructions had to be executed at recall, there were no significant differences in performance between the three conditions (all *p* > .140). Regarding the main effect of recall, Bonferroni-corrected planned comparisons (paired *t* tests) established that accuracy was higher for action than for verbal recall across all presentation conditions: *t*(59) = 6.586, *p* < .001, *d* = 1.09 for no enactment, *t*(59) = 2.531, *p* = .015, *d* = .34 for enactment, and *t*(59) = 5.735, *p* < .001, *d* = .92 for shadowing.

### Discussion

There were three key findings from this experiment. First, there was an advantage for action over verbal recall in all conditions, but the magnitude of this effect was reduced when participants enacted the instructions during their initial presentation. This replicates the findings from Experiment 1 and those reported with adults by Allen and Waterman (2015), and suggests that there is little gain to be had from action-based recall following action-based encoding. This point will be discussed further in the general discussion.

Second, verbally shadowing spoken instructions did not enhance recall compared with listening to the commands only. This finding rules out the possibility that the motoric enactment effect found in both experiments simply reflects a mnemonic benefit of the physical generation of an action (in this case, verbal articulation) that corresponds to the content of the instructions. It is consistent with the proposal that this enactment effect, like the SPT phenomenon, arises from a distinctive property of motor actions (e.g., Engelkamp, 2001; Engelkamp & Zimmer, 1994). Finally, the presentation times were equivalent in the physical enactment and verbal shadowing conditions. Differences in performance for enacted and shadowed sequences are therefore not the result of differences in encoding time.

## General discussion

These experiments have generated three novel findings relating to the benefits of physical performance of spoken sequences of instructions. First, beneficial effects of both enactment at encoding and action at recall were observed, for the first time, in typically developing children. This adds to emerging evidence indicating that planning for or implementing a set of physical actions facilitates working memory performance (Allen & Waterman, 2015; Gathercole et al., 2008; Koriat et al., 1990; Yang et al., 2014). The mnemonic advantage of action at recall was greatly reduced when combined with enactment at encoding. This pattern is consistent with Allen and Waterman’s (2015) findings with adults, indicating that the two phenomena have a common origin. What might this be? We propose that when executing physical actions during encoding or planning for action recall, children may actively construct action plans that incorporate spatio-motoric information and representations of intended movements (Choudhury, Charman, Bird, & Blakemore, 2007; Koriat et al., 1990; Wolpert & Ghahramani, 2000), which are held in a specialized motor store in working memory (see also Smyth & Pendleton, 1989, 1990). The benefits to recall afforded by action are therefore provided by an additional source of information in working memory that supplements information maintained in the phonological loop and visuospatial sketchpad. In line with this interpretation, planning to execute action sequences did not enhance children’s recall when the instructions had already been enacted at presentation because the executed action sequence was already represented in the motor store. One possible concern is that the effect of action-based recall is diminished when combined with instruction at presentation simply because baseline performance is higher and hence has reduced capacity for further gain. Although performance is certainly not at ceiling level, this possibility cannot at present be ruled out.

This interpretation is compatible with the broader concept that cognition is grounded within sensation and action that has been widely adopted in the study of perception and action (for a review, see Gentsch, Weber, Synofzik, Vosgerau, & Schütz-Bosbach, 2016). For example, Clark (1999) has described how action-oriented representations might provide efficient forms of computation derived either from actual or simulated sensing and acting. This approach is in principle consistent with the modular domain-specific storage systems of the Baddeley (2000) working memory model, in which sensory and motor resources could be conceived as being recruited to “embody” information for temporary storage (Wilson, 2001, 2002). This could be the source of sensorimotor coding both in simulated (in the case of planning for enacted recall) and actual (enactment during encoding) action in the present paradigm of serial recall of action sequences.

Although the mnemonic advantage attributed to action recall was reduced following enactment during encoding, recall was most accurate when enactment during encoding was followed by action at recall across both experiments. This is consistent with the principles of transfer appropriate processing that performance will be enhanced when the procedures required at test match those employed during encoding (e.g., Morris, Bransford, & Franks, 1977; Roediger, Gallo, & Geraci, 2002). It is also in line with encoding specificity, which provides a framework for understanding how contextual information affects recall performance and states that memory is most effective when information available at encoding is also present at retrieval (e.g., Baddeley, 1999; Tulving, 1983).

Second, neither verbally shadowing individual steps in the spoken instruction sequence (Experiment 2) nor additionally reading aloud their printed forms (Experiment 1) enhanced the ability to remember the instructions, irrespective of the method of recall. This suggests that the beneficial impact of performing instructions at presentation is not simply a consequence of exposure to two different representational forms (because reading the instructions did not lead to a comparable improvement in performance) or that the generation of any form of motor response is critical (because saying the instructions did not help whereas performing them did). In the latter case, equivalency in terms of the time taken to enact and vocalize each instruction step during presentation rules out an explanation of the superior recall of enacted instructions being related to additional encoding time. These findings are consistent with accounts that attribute enactment effects in longer term SPT paradigms to the rich multimodal nature of the representations generated by physical performance (e.g. Bäckman et al., 1986; Bäckman & Nilsson, 1984, 1985; Roediger & Zaromb, 2010). Here we have demonstrated that the same applies to immediate memory paradigms.

Finally, we found that the action advantage at recall was eliminated when each step of the instructions was displayed in printed form immediately after it had been spoken in Experiment 1. One speculative explanation is that the immediate presentation of text in the orthographic condition, the saccadic eye movements associated with reading, and the inevitable shift of attentional focus away from the object display to the laptop screen may have prevented the construction of motoric representations of the planned sequence. It may also have reinforced the use of a verbal encoding strategy.

The robust and specific advantage to children of performing spoken instructions demonstrated in these experiments may have practical relevance for classroom practice. It has been proposed that impairments in abilities to follow verbal instructions given by the teacher may impair academic progress (Engle et al., 1991; Gathercole, Lamont, & Alloway, 2006). Physical engagement both at the time that instructions are presented and at recall may provide a useful means of boosting the accuracy of remembering instructions over short as well as longer periods. Indeed, by recruiting an additional source of mnemonic benefit in the form of a temporary motor store, children may be able to minimize the adverse consequences of weak verbal memory skills. In this way, incorporating physical engagement within curricular activities may have the potential to accelerate learning.
